# Graphene-based versus alumina supports on CO_2_ methanation using lanthanum-promoted nickel catalysts

**DOI:** 10.1007/s11356-023-26324-7

**Published:** 2023-03-16

**Authors:** David Méndez-Mateos, V. Laura Barrio, Jesús M. Requies, Miryam Gil-Calvo

**Affiliations:** School of Engineering (UPV/EHU), Plaza Ingeniero Torres Quevedo 1, 48013 Bilbao, Spain

**Keywords:** CO_2_ emission reduction, Methanation, Graphene oxide, Nickel catalysts, Alumina

## Abstract

The valorization of CO_2_ as a biofuel, transforming it through methanation as part of the power-to-gas (P2G) process, will allow the reduction of the net emissions of this gas to the atmosphere. Catalysts with 13 wt.% of nickel (Ni) loading incorporated into alumina and graphene derivatives were used, and the effect of the support on the activity was examined at temperatures between 498 and 773 K and 10 bar of pressure. Among the graphene-based catalysts (13Ni/AGO, 13Ni/BGO, 13Ni/rGO, 13Ni-Ol/GO, 13Ni/Ol-GO, and 13Ni/Ol-GO Met), the highest methane yield was found for 13Ni/rGO (78% at 810 K), being the only system comparable to the catalyst supported on alumina 13Ni/Al_2_O_3_ (89.5% at 745 K). The incorporation of 14 wt.% of lanthanum (La) into the most promising supports, rGO and alumina, led to nickel-support interactions that enhanced the catalytic activity of 13Ni/Al_2_O_3_ (89.5% at lower temperature, 727 K) but was not effective for 13Ni/rGO. The resistance against deactivation by H2S poisoning was also studied for these catalysts, and a fast deactivation was observed. In addition, activity recovery was impossible despite the regeneration treatment carried out over catalysts. The resistance against deactivation by H2S poisoning was also studied for these catalysts, observing that both suffered a rapid/immediate deactivation and which in addition/unfortunately was impossible to solve despite the regeneration treatment carried out over catalysts.

## Introduction

The amount of carbon dioxide in the atmosphere is increasing due to anthropogenic causes, reaching values of 407.4 ppm in 2018 (Blunden and Arndt 2019) and consequently increasing the average global temperature. One of the main causes of these high values is the combustion of fossil fuels, being responsible for three quarters of the total CO_2_ emissions (Khapre et al. 2020). Therefore, nowadays the main objective is focused on reducing the amount of CO_2_ present in the atmosphere. Some of the strategies that are currently proposed are based on carbon capture to obtain bioenergy (BECCS) (Choi et al.) or through the capture by adsorption of CO_2_ in microporous systems and its transformation for the utilization (CCU) (Kim et al. 2019; Nocito and Dibenedetto 2019). Among the applications of captured CO_2_, the production of fuel, obtaining fuel stands out, in order to meet the energy demand without producing more CO_2_ as in the case of methanol (MeOH) (Xiong et al. 2019; Fang et al. 2019), dimethyl carbonate (DMC) (Marciniak et al. 2019; Xuan et al. 2019), dimethyl ether (DME) (Ren et al. 2019), solid carbonate (Favre et al. 2009), and methane (Hu et al. 2019). In this sense, the catalytic hydrogenation of CO_2_ constitutes an interesting way to produce methane from CO_2_. This path requires hydrogen, which is already an efficient fuel, but with more disadvantages in terms of storage and transport than methane, due to its lower energy density (Salomone et al. 2019). Hydrogen production is one of the challenges in the recovery of CO_2_ from the atmosphere, and the most sustainable method to get it is the electrolysis of water using electricity from wind or solar energy. Power-to-gas (P2G) is a technology that transforms surplus electrical power from renewable energy sources into methane as a gas fuel that can be stored or distributed through the natural gas network (Bassano et al. 2019). P2G process encompasses two principal stages: the electrolysis of water to obtain H_2_ and the use of this H_2_ in methanation of CO_2_ captured from the atmosphere or industrial gaseous effluents to obtain CH_4_. The hydrogenation of CO_2_ which was studied by Sabatier is an exothermic reaction, as shown in Eq. 1 (Shiva and Himabindu 2019).

1 $${\mathrm{CO}}_{2}+4{\mathrm{H}}_{2}\rightleftarrows 2{\mathrm{H}}_{2}\mathrm{O}+{\mathrm{CH}}_{4} \Delta {\mathrm{H}}_{298\mathrm{ K}}^{0}=-165\mathrm{ kJ}{\cdot \mathrm{mol}}^{-1}$$

This reaction is the combination of the Reverse Water Gas Shift (RWGS) (Eq. 2) and the CO methanation (Eq. 3). Due to the exothermicity of the reaction and according to the thermodynamic laws, as the temperature and pressure increase, the yield of the reaction decreases (Bassano et al. 2019). That is exactly why the choice of a suitable catalyst and support has gained great importance, to achieve a proper dispersion of active centers and avoid hot spots.

2 $${\mathrm{CO}}_{2}+{\mathrm{H}}_{2}\rightleftarrows \mathrm{CO}+{\mathrm{H}}_{2}\mathrm{O }\Delta {\mathrm{H}}_{298\mathrm{ K}}^{0}=41\mathrm{ kJ}{\cdot \mathrm{mol}}^{-1}$$

3 $$\mathrm{CO}+3{\mathrm{H}}_{2}\rightleftarrows {\mathrm{CH}}_{4}+{\mathrm{H}}_{2}\mathrm{O }\Delta {\mathrm{H}}_{298\mathrm{ K}}^{0}=-206\mathrm{ kJ}{\cdot \mathrm{mol}}^{-1}$$

According to the literature, not only noble metals such as ruthenium (Ru), rhodium (Rh), and palladium (Pd) have been found effective in CO_2_ methanation but also transition metals, such as nickel (Ni), were also successfully employed (Hu et al. 2019; Garcia-Garcia et al. 2016). Nickel-based catalysts are commonly reported because of their low price, availability, and competitive activity, proposing them as an attractive alternative to noble metals (Hu et al. 2019; Garcia-Garcia et al. 2016). Transition and noble metals are mainly supported over metal oxides such as Al_2_O_3_, SiO_2_, TiO_2_, CeO_2_, Y_2_O_3_, and zeolites and even, in new works, reduced graphene oxide (rGO) (Hu et al. 2019; Garbarino et al. 2015; Beuls et al. 2012). Among the cited supports, γ-alumina is the most typical oxides because its high surface area leads to a better dispersion of the active metal on the surface (Mihet and Lazar 2018; Kuzmenko et al. 2019; Swalus et al. 2012). Recent research has shown that modification of the γ-alumina support in nickel-based catalysts for the methanation reaction enhances the activity, especially at low temperatures (Garcia-Garcia et al. 2016; Liang et al. 2019). In the case of modifying alumina support with different amounts of lanthanum (0, 4, 14, and 37 wt.%), it was concluded that the methane yield increases at low temperatures up to a lanthanum loading of 14 wt.% (Garbarino et al. 2019). In Table 1 are summarized some of the results of methanation reaction.

**Table 1:** Summary of the catalytic activity of methanation reaction

Catalyst	CH_4_ yield	CO_2_ conversion	CH_4_ selectivities	Authors
Ni/Ac	60.0% (623 K)	60.1% (723 K)		Hu et al. 2019
Ni/γ-Al_2_O_3_	62.0% (623 K)	63.1% (673 K)		Hu et al. 2019
Ni/rGO	78.0% (623 K)	78.4% (623 K)		Hu et al. 2019
Ni-Ce/rGO	82.3% (623 K)	85.4% (623 K)		Hu et al. 2019
Ni-Ce/Ac	73.0% (673 K)	75.2% (623 K)		Hu et al. 2019
Ni-Ce/γ-Al_2_O_3_	78.0% (623 K)	78.7% (673 K)		Hu et al. 2019
13Ni/Al_2_O_3_	71.0% (673 K)			García–García et al. 2016
13Ni-1Rh/Al_2_O_3_	69.0% (700 K)			García–García et al. 2016
13Ni-1Ru/Al_2_O_3_	67.0% (750 K)			García–García et al. 2016
13Ni/6Ce-Al_2_O_3_	74.0% (643 K)			García–García et al. 2016
13Ni-1Rh/6Ce-Al_2_O_3_	68.0% (673 K)			García–García et al. 2016
3Ru/Al_2_O_3_	82.0% (623 K)	86.0% (623 K)		Garbarino et al. 2015
20Ni/Al_2_O_3_	71.0% (623 K)	78.0% (623 K)		Garbarino et al. 2015
Ni/γ-Al_2_O_3_		90.0% (673 K)	≥ 95.0% (673 K)	Mihet and Lazar 2018
Ni-Pt/γ-Al_2_O_3_		83.4% (673 K)	≥ 95.0% (673 K)	Mihet and Lazar 2018
Ni-Pd/γ-Al_2_O_3_		90.6% (673 K)	≥ 95.0% (673 K)	Mihet and Lazar 2018
Ni-Rh/γ-Al_2_O_3_		64.7% (673 K)	≥ 95.0% (673 K)	Mihet and Lazar 2018
Ni/Ac	4.0% (398 K)	4.0% (398 K)		Swalus et al. 2012
Rh/γ-Al_2_O_3_	50.0% (398 K)	50.0% (398 K)		Swalus et al. 2012
Ni/Al_2_O_3_	18.0% (573 K)	72.0% (873 K)		Liang et al. 2019
Sr-Ni/Al_2_O_3_	70.0% (573 K)	70.0% (573 K)		Liang et al. 2019
Ba-Ni/Al_2_O_3_	80.0% (573 K)	80.0% (573 K)		Liang et al. 2019
NiØLA	80.0% (623 K)			Garbarino et al. 2019
Ni4LA	86.0% (673 K)			Garbarino et al. 2019
Ni14LA	92.0% (623 K)			Garbarino et al. 2019
Ni37LA	82.0 (623 K)			Garbarino et al. 2019

Graphene, owing to its physicochemical characteristics, has proven to be an interesting material for medical applications (Song et al. 2020; Xia et al. 2019), sensors (chemical, biological, gas, or electrochemical among others) (Saleh and Fadillah 2019; Taniselass et al. 2019; Seekaew and Wongchoosuk 2019; Afsari and Sarraf 2020; Kumar et al. 2020; Reddeppa, et al. 2020; Beitollahi et al. 2020), electrocatalytic applications (Urbanczyk et al. 2019; Simanjuntak et al. 2020), photovoltaic applications (Ansari et al. 2019; Mehmood et al. 2020), energy storage applications (Korkmaz and Kariper 2020; Wang et al. 2020), or reinforcement of materials (Cui et al. 2019; Rahman et al. 2018). Recently, these promising properties of graphene have been applied to support catalysis of different reactions (Lei et al. 2019; Masteri-Farahari et al. 2019; Ahmad et al. 2017). Hybridization of the orbitals of the carbon atom for the formation of sp^2^ bonds results in the formation of 3 σ bonds and one delocalized π bond, drawing a double bond. In this way, the angle obtained between the bonds is 120° that gives the graphene structure a hexagonal or honeycomb shape. Caused by this peculiar two-dimensional structure, with delocalized π bonds, a conduction band and a valence band are generated which are in turn responsible for most of the conduction properties of the graphene (Warner et al. 2013). The ability to dissipate the heat originated from the exothermic methanation//Sabatier reaction, added to the large surface area and mechanical strength of graphene, makes it a support of great interest for this application. Because it contains less hydroxyl, epoxy, and carboxyl side groups, its centers are able to act as anchoring or nucleation centers, which could improve the dispersion of the metallic nanoparticles. In addition, the cost-effectiveness and easy scale-up potential of GO make it a promising support (Warner et al. 2013). Reduced graphene oxide (rGO) is another product derived from graphene oxide, obtained by thermal, chemical, or electrochemical reduction of graphene oxide. GO presents a complex chemical structure due to its irregular density of oxygen group defect. These defects cause insulator characteristic in GO. Although the reduction of GO (rGO) could increase 3–9 times the conductivity (Trikkaliotis et al. 2021), GO also present superb photoluminescence; that is its applicability in biosensing and photoelectronics. Due to its characteristic, GO is used as catalytic support in many photocatalytic processes (Mousari-Salehi et al. 2023, Sachdeva 2020). Due to the functional groups on the surface of GO, GO is also effective for immobilizing various catalytically active species (Sachdeva 2020). Like GO, rGO can be scaled up and manufactured on kilogram scale (Rowley-Neale et al. 2018). During the reduction step to obtain rGO, despite the fact that a large part of the oxygen contained in its structure is eliminated, some functional groups of oxygen are maintained, so the interaction between the nanoparticles and the structure of the support is still favored, which could increase its dispersion and stability (Hu et al. 2019). Another graphene-derived material/support that can improve GO characteristics was obtained by the incorporation of an aminated group into the structure of graphene oxide (Hu et al. 2019). The aminated group reacts with oxygen atoms present in the graphene base layer, causing its replacement. The effect of this addition depends on the characteristics of the chain length and the linked amino group. In the case of long chain compounds, such as oleylamine, it could give hydrophobic character to the resulting aminated graphene oxide (Tang et al. 2016). In several works (Narwade et al. 2019; Primo et al. 2019; Ismail et al. 2015; Mateo et al. 2018), metals have been also incorporated into rGO supports with improved yields in different reactions.

In the present work, the effect of graphene-based materials (provided by Graphenea), with 13 wt.% of nickel, on CO_2_ methanation was studied and compared with catalysts supported on alumina. Among graphene materials on the one hand, commercial reduced graphene oxide (13Ni/rGO) and graphene oxide reduced with methylamine borane (MeAB) (13Ni/BGO) were analyzed. On the other hand, commercial aminated graphene oxide (13Ni/AGO) and oleylamine aminated graphene oxide prepared by three different procedures (13Ni-Ol/GO, 13Ni/Ol-GO and 13Ni/Ol-GO (Met)) were analyzed. Finally, the best of these catalysts, which turned out to be 13Ni/rGO, was modified with 14 wt.% of lanthanum (13Ni/14La-rGO). Then, it was compared with catalysts supported on alumina (13Ni/Al_2_O_3_ and 13Ni/14La-Al_2_O_3_), to determine the most efficient system for the hydrogenation of CO_2_.

## Experimental

### Catalyst preparation

The catalyst 13Ni/AGO was prepared by wet impregnation method, mixing 2 g of aminated graphene oxide (AGO) (provided by Graphenea) with 1.48 g of nickel (II) nitrate hexahydrate (99.999%; Sigma-Aldrich) and 500 ml of ethanol (100%; Sigma-Aldrich). In order to incorporate the nickel into the AGO pore structure, the solution was sonicated for 2 h. Then, the solvent was removed in a rotary evaporator (Heidolph Laborota 4000) at 323 K under vacuum. The catalyst precursor was dried in an oven for 2 h at 393 K. Finally, in a thermogravimetric analyzer (TGA/SDTA851 Mettler Toledo) under N_2_ atmosphere, the calcined catalyst 13Ni/AGO was obtained. This calcination program/sequence consisted of 4 stages with a temperature ramp of 1 K min^−1^ followed by an isothermal step for 1 h at each temperature, except for the third one. The temperature ranges of each stage were set at 273–378 K (water removal), 378–453 K (heating 1), 453–493 K (heating 2), and 493–673 K (heating 3).

The reduction of graphene oxide with methylamine borane (MeAB) to obtain 13Ni/BGO was carried out in two stages (Shen et al. 2015). The first step consisted in the preparation of methylamine borane. For this purpose, 11.35 g of sodium borohydride (> 98.0%; Sigma-Aldrich) and 20.26 g of methylamine hydrochloride (> 98.0%; Sigma-Aldrich) were dissolved in 600 ml of tetrahydrofuran (THF) (> 99.9%; Honeywell) in a round bottom flask connected to a condenser and vigorously stirred. The reaction was carried out under N_2_ atmosphere at room temperature for 12 h. The solution was then filtered in a Büchner funnel by vacuum filtration, and the filtrate was concentrated under vacuum at room temperature in a rotary evaporator. The product was purified with methanol (> 99.9%; Sigma-Aldrich). The second stage comprised the synthesis of 13Ni/BGO. 3 g of GO (100%; Graphenea) was dissolved in 580 ml of Milli-Q water in a round bottom flask. After, 2.22 g of nickel (II) nitrate hexahydrate (99.999%; Sigma-Aldrich) was added and the solution was sonicated for 2 h. Subsequently, 400 ml of Milli-Q water containing 27 g of MeAB was added to the solution under vigorous stirring. The obtained mixture was stirred overnight in a rotary evaporator before being concentrated at 313 K under vacuum. The collected concentrate was dried in an oven at 393 K for 2 h and was calcined in the TGA in N_2_ atmosphere, following the program given above in the preparation of AGO, which resulted in the sample labeled as13Ni/BGO.

Oleylamine aminated catalysts were prepared by three different procedures to obtain three different catalysts, depending on when the nickel salt is added and if a purification step is included to remove the oleylamine excess (Tang et al. 2016). The first one consisted in the mixture of GO and oleylamine (70%; Sigma-Aldrich) in the same proportion (3 g) dissolved in 500 ml of ethanol with stirring for 30 min. The solution was sonicated for 2 h, and the solvent was evaporated in a rotary evaporator at 323 K and vacuum. Once the sample was dried in an oven at 393 K for 2 h, the cooled solid was mixed with the appropriate amount of nickel salt and was dissolved in 500 ml of ethanol, repeating the previous procedure to obtain the 13Ni/Ol-GO. The second oleylamine aminated catalyst was prepared following the same procedure but including/integrating a methanol purification step once the sample was dried and cooled, before introducing nickel, and this catalyst was named 13Ni/Ol-GO (Met). For the synthesis of the third type of these catalysts, 13Ni-Ol/GO, the nickel salt was initially mixed with the oleylamine and ethanol, before proceeding as in the previous cases.

The catalysts supported on rGO were also prepared by wet impregnation in two successive steps, mixing 2 g of rGO with 0.90 g of lanthanum salt (lanthanum (III) nitrate hydrate, 99.9%; Sigma-Aldrich) and 500 ml of ethanol. With the aim of incorporating lanthanum into the rGO pore structure, the solution was sonicated for 2 h. Then, the solvent was removed in a rotary evaporator at 323 K under vacuum. The modified support was dried in an oven for 2 h at 393 K and finally calcined in a thermal gravimetric analysis (TGA) employing the same temperature sequence of the previous cases. To incorporate the nickel on the rGO and La-rGO support, the procedure was similar to that followed for lanthanum. 1.76 g of nickel salt was added to the prepared support, and it was dissolved in 500 ml of ethanol and sonicated. After evaporation of the solvent in the rotary evaporator (under vacuum at 323 K), it was dried in an oven for 2 h at 393 K and calcined with the temperature program of the previous cases in TGA, obtaining the final catalysts 13Ni/rGO and 13Ni/14La-rGO.

The catalysts supported on alumina were prepared by wet impregnation in two successive steps. 5 g of γ-Al_2_O_3_ (Merck) and 2.25 g of lanthanum salt were mixed to obtain the 14 wt.% of La. After that, 20 ml of Milli-Q distilled water was added to the previous mixture and the pH value of the solution was adjusted to 8.5 (Trueba and Trasatti 2005; Franks and Meagher 2003). The solution was stirred overnight in a rotary evaporator, and the solvent was eliminated by heating at 338 K under vacuum. The support obtained was dried in an oven at 373 K during 2 h and calcined at 673 K in air atmosphere for 2 h with a ramp of 1 K/min. Finally, nickel was incorporated into the bare alumina and lanthanum-modified support by wetness impregnation, thus obtaining 13Ni/Al_2_O_3_ and 13Ni/14La-Al_2_O_3_ catalysts.

Eventually, the calcined catalysts were pressed and sieved to the desired particle size range (0.42 mm < *d*_p_ < 0.50 mm). This particle size (*d*_p_) was chosen in order to avoid reagents bypassing near the wall, accordingly to keep an internal pipe diameter-to-particle size ratio higher than 10 (Focus Catal 2016).

As it has been mentioned, and as a summary, the catalysts prepared were named, according to their nominal composition, as follows: 13Ni/AGO, 13Ni/BGO, 13Ni/Ol-GO, 13Ni/Ol-GO (Met), 13Ni-Ol/GO, 13Ni/rGO, 13Ni/14La-rGO, 13Ni/Al_2_O_3_, and 13Ni/14La-Al_2_O_3_.

### Catalyst characterization

The N_2_ adsorption method was employed to evaluate textural properties of the catalysts in the Autosorb 1C-TCD, after degassing the samples at 573 K for 3 h. The specific surface area was determined using the Brunauer–Emmett–Teller (BET) equation, and pore volume and pore sizes were calculated by the Barrett-Joyner-Halenda (BJH) method.

The Inductively Coupled Plasma Optical Emission Spectroscopy (ICP-OES) analyzer in a Perkin–Elmer Optima 3300DV was employed to determine the composition of the catalysts. Prior to the analysis, the catalyst was disaggregated in an acid solution (75% HCl and 25% HNO_3_, in volume). Their characteristic wavelength emission bands identified the composition and chemical elements. The elemental concentrations of carbon, hydrogen, and nitrogen present in the catalysts were determined in a LECO TruSpec CHN by heating up 30–100 mg of the catalysts up to 1223 K for 300 s under O_2_ flow.

The structural properties of the catalysts were investigated by X-Ray diffraction (XRD) and measurements were performed using a PANalytical X’Pert Pro diffractometer with a CuK_α_ radiation ($\lambda =0.15418$ nm) operating at 40 kV and 30 mA. The collected XRD patterns were matched using the JCPDS database, and crystalline phases were identified. Moreover, the average crystallite size was estimated by the Scherrer equation (Cheng et al. 2017). The 2θ position of the diffraction peaks of the XRD patterns was determined by X-ray diffraction. The peaks were identified with the help of database Joint Committee on Powder Diffraction Standards (JCPDS) for the fresh and used catalysts.

Temperature programmed reduction (TPR) examined the reducibility of the catalysts with hydrogen in a Micromeritics AutoChem II Instrument equipped with a thermal conductivity detector (TCD). The TPR profiles were recorded heating 10 mg of the sample from 323 to 773 K for rGO-supported catalysts and 50 mg from 323 to 973 K for alumina-supported catalysts, at a ramp rate of 10 K/min. Prior to the analysis, the sample was pretreated in Ar stream (30 Nml/min) from room temperature to 473 K for 1 h. Later, the sample was cooled down to 323 K and Ar was replaced by 5 vol.% H_2_/Ar (45 Nml/min) stream to start the reduction.

X-ray photoelectron spectroscopy (XPS) was performed on a SPECS (Berlin, Germany) system equipped with a Phoibos 150 1D-DLD analyzer and an Al Kα (1486.6 eV) monochromatic radiation source with electron output angle of 90°. XPS measurements allowed the study of the species present on the surface of the catalyst.

High-resolution scanning and transmission electron microscopy (HR-STEM or only STEM) was employed to analyze the morphology of the catalysts. The measurements were carried out in a Schottky X-FEG (FEI Titan Cubed G2 60–300) transmission and scanning-transmission electron microscopy with resolution ≤ 0.136 nm at 300 kV and resolution in energy ≤ 0.3 eV. Additionally, the instrument was equipped with EDX Super-X for chemical analysis.

### Activity tests

The CO_2_ hydrogenation activity was tested by measuring methane yield in a stainless steel fixed-bed reactor of 32 cm length, with an outside diameter of ¼″ within a bench-scale plant (PID Eng&Tech) at 10 bar and a temperature range of 498–773 K.

**Fig. 1:**
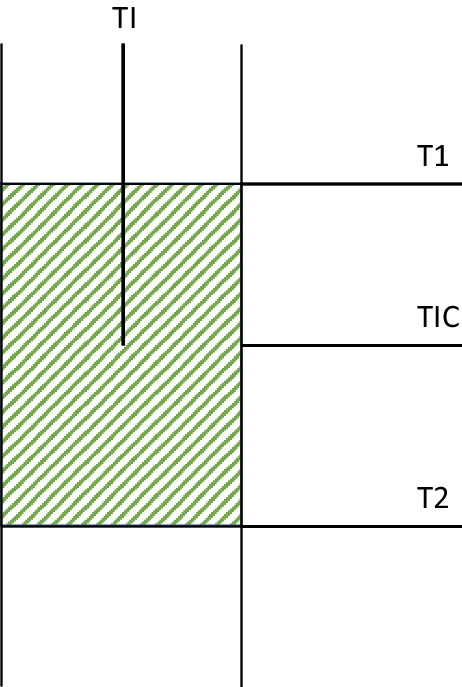
Position diagram of thermocouples in the system. Control thermocouple (TIC), catalytic bed thermocouple (TI), and test thermocouples (T1 and T2)

Typically, the reactor was loaded with 60–200 mg of catalyst diluted with inert SiC in order to minimize possible thermal gradients in the catalytic bed (weight_catalyst_/weight_SiC_ = 1:4.5). Furnace temperature was adjusted to maintain the catalytic bed under isothermal condition using four thermocouples as showed in Fig. 1. The reactor was heated with a rate of 10 K/min under N_2_ flow until the reaction temperature. The catalytic activity was studied with a temperature interval of 25 K from 498 to 573 K, and with an interval of 50 K from 573 to 773 K. The first step was the catalyst activation, reducing the catalyst with a ratio of N_2_ (99.999%, Air Liquide) to H_2_ (99.999%, Air Liquide) equal to 3:1 (65 Nml/min of H_2_) at 673 K during 4 h. Once the catalyst was activated, H_2_ and CO_2_ (99.98%, Air Liquide), the methanation reaction gases, were fed in a volume ratio H_2_:CO_2_ of 4:1. In such way, the reaction (Eq. 1) was performed at a Weight Hourly Space Velocity (WHSV) of 128.75 g_feed_/(g_cat_·h).

The reaction was stabilized at each temperature for 120 min, analyzing the products separated in a Peltier condenser. On the one hand, after each reaction temperature, condensed water was collected and weighted. On the other hand, the gas stream was analyzed in an online MicroGC Varian CP-4900 equipped with a high-sensitivity TCD and two columns (10 m Molecular Sieve 5 and 10 m Poraplot Q) and, in a complementary way, the flow was also measured with a mass flowmeter. The parameter used to evaluate the catalytic activity was the yield of methane, calculated/determined by the following equation:

4 $$\mathrm{CH}4\mathrm{ yield}: {\upeta }_{{\mathrm{CH}}_{4}}=\mathrm{mol }{\mathrm{CH}}_{4}^{\mathrm{out}}/\mathrm{mol }{{\mathrm{CH}}_{4}^{\mathrm{out}}}_{\mathrm{stoichiometric}}\cdot 100$$

In Eq. 4, ${\eta }_{{\mathrm{CH}}_{4}}$ symbolize the methane yield; mol CH_4_^out^ symbolize the flow rate of the methane out of the reactor; mol CH_4_^out^_stoichiometric_ symbolize the theoretical flow rate of the methane out of the reactor, based on the stoichiometry of the methanation/Sabatier reaction. Thus, if all CO_2_ fed was converted to CH_4_, the value of ${\eta }_{{\mathrm{CH}}_{4}}$ would be 100%. It should be mentioned that all the experiments were repeated at least twice in order to ensure the reproducibility of the obtained results.

## Results and discussion

### Catalyst characterization

#### Chemical composition

The metallic content (nickel and lanthanum) of the catalysts was determined by ICP-OES and the corresponding data, with the nominal values in brackets, are collected in Table 2.

**Table 2:** Summary of the contents of Ni and La, specific area, particle size of Ni, and relative nickel dispersion of the samples

Catalyst	Theoretical content		Content by ICP		*S*_BET_^a^ (m^2^/g)	*V*_P_^b^ (cm^3^/g)	*d*_P_^c^ (nm)	*d*_XRD_ (nm)^d^	
	Ni	La	Ni	La				Reduced	Used
13Ni/AGO	13	0	18.5	0	–	–	–	–	–
13Ni/BGO	13	0	1.3	0	–	–	–	–	–
13Ni-Ol/GO	13	0	27.4	0	–	–	–	–	–
13Ni/Ol-GO	13	0	52.9	0	–	–	–	–	–
13Ni/Ol-GO (Met)	13	0	38.8	0	–	–	–	–	–
rGO	0	0	0	0	619	1.96	3.8	–	–
13Ni/rGO	13	0	12.9	0	452	1.30	3.8	8	6
13Ni/14La-rGO	13	14	15.6	11.4	258	0.70	3.8	80*	7
γ-Al_2_O_3_	0	0	0	0	202	0.81	7.7	–	–
13Ni/Al_2_O_3_	13	0	13.9	0	180	0.55	7.2	5	10
13Ni/14La-Al_2_O_3_	13	14	15.4	11.6	92	0.14	3.6	5	15

^a^The surface area was calculated by the BET equation

^b^BJH desorption pore volume

^c^BJH desorption average pore diameter

^d^Calculated from Ni (111) plane using the Scherrer equation

Catalysts containing amine groups showed higher nickel content, because the decomposition of amine group leads to a reduction of the support mass and consequently the contribution of the nickel loading increases. It should be noted that, in the 13Ni/BGO catalyst, the reducing agent was incorporated into the catalyst instead of being removed in the cleaning/purification steps, and this contribution was not considered for the composition calculations. That is why in this case the amount of incorporated metal was so low, 1.5 wt.% instead of 13 wt.%. This fact implies that it will be necessary to use a larger amount of catalyst to keep comparable operation conditions, namely, active metal loading and WHSV.

Regarding catalysts supported on alumina and rGO, the theoretical Ni content was set at 13 wt.%, and for lanthanum-modified supported catalysts, a content of 14 wt.% La was desired. The catalyst 13Ni/Al_2_O_3_ showed an experimental nickel loading slightly higher compared to the nominal value, which is in agreement with García-García et al. (2016) results over Ni catalyst supported on γ-Al_2_O_3_. However, the experimental content of nickel was higher than the target composition in the presence of lanthanum, probably due to some losses in support weight, associated to the reduction of its oxygen content. The nickel content analyzed in the rGO-supported catalysts showed a value close to the nominal value.

#### BET measurements

**Fig. 2:**
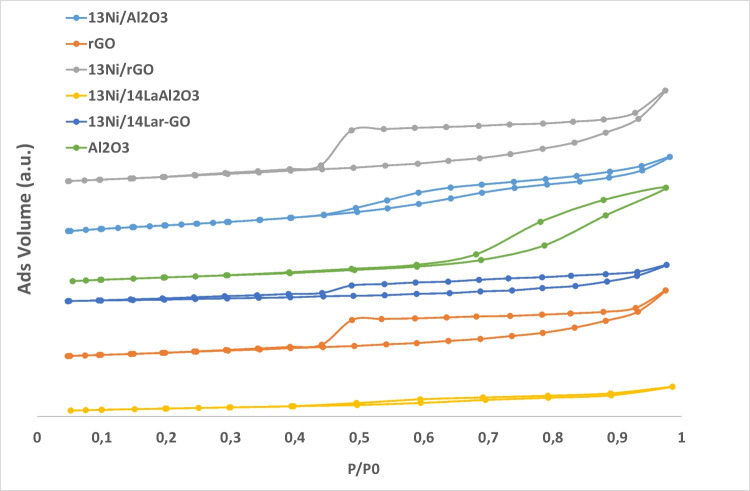
Isotherm of N_2_ adsorption and desorption

The physical properties of the supports and the nickel catalysts were examined by nitrogen adsorption–desorption isotherms. All the samples exhibited IV-type isotherms (Fig. 2) with a remarkable H2-type hysteresis loop, thus displaying a typical curve associated with the presence of mesoporous structure due to the presence of “ink-bottle” or cylindrical channels (Sing and Williams 2004). Table 2 summarizes the specific surface area (*S*_BET_), pore volume (*V*_P_), and average pore size (*d*_P_) of the catalysts, as well as the information corresponding to bare alumina and rGO support materials.

Regarding bare supports, rGO had a markedly larger surface area (619 m^2^/g) than alumina (202 m^2^/g), which could later lead to a better Ni dispersion (Hu et al. 2019). With the addition of nickel, both supports underwent a significant loss of surface area and pore volume (from 619 to 452 m^2^/g and 1.96 to 1.30 cm^3^/g for rGO and from 202 to 180 m^2^/g and 0.81 to 0.55 cm^3^/g for alumina, respectively), owing to the partial blockage of their small pores (Bang et al. 2012). The incorporation of a support modifier, namely, lanthanum, contributed negatively to the blocking effect of the pores. As a consequence, La-modified catalysts (13Ni/14La-rGO and 13Ni/14La-Al_2_O_3_) suffered not only a considerable reduction of the total surface area and pore volume but also a decrease in the pore size in the case of alumina-supported catalyst (Garbarino et al. 2019).

#### X-ray diffraction

**Fig. 3:**
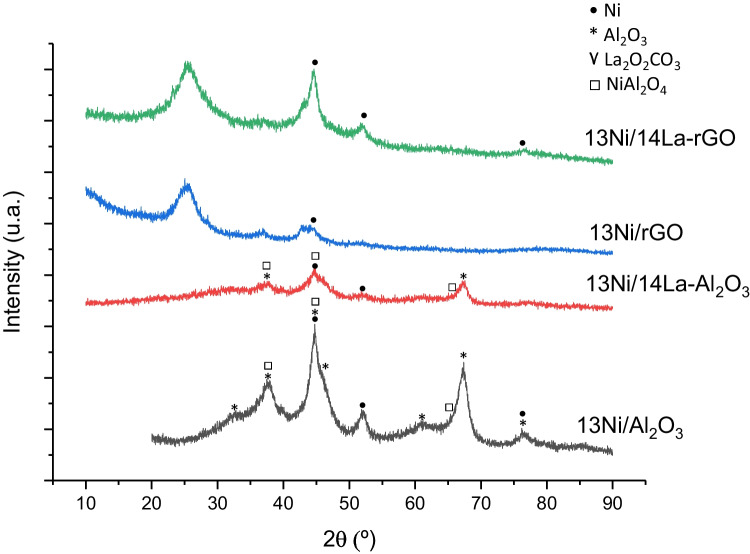
XRD patterns of bi- and monometallic freshly reduced catalysts supported over Al_2_O_3_ and rGO

**Fig. 4:**
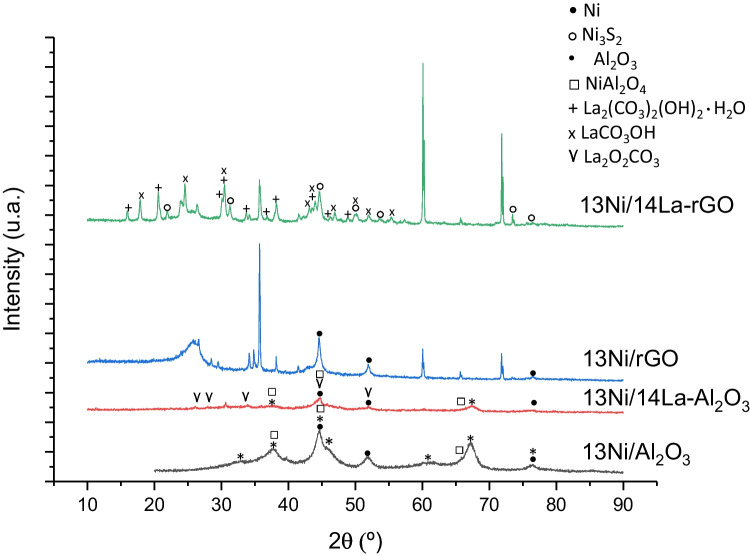
XRD patterns of bi- and monometallic used catalysts supported over Al_2_O_3_ and rGO

Figures 3 and 4 show the XRD patterns of the fresh and used rGO and alumina-supported catalysts, respectively, being possible the identification of the main crystalline species. Figure 3 shows the crystalline species in a reduced state, prior to the use of the catalysts in reaction. The γ-Al_2_O_3_ support exhibits diffraction peaks at 37.5°, 44.5°, and 67.4° mainly, corresponding to the alumina crystal plane of (331), (400), and (440), respectively (Paglia 2004; Segal et al. 2018). Peaks at 19.7° (111), 34° (220), 57° (422), 61° (511), and 85° (444) can also be used for the identification of the alumina, although they were more difficult to detect especially in the presence of metals (Paglia 2004; Segal et al. 2018). The diffraction peaks of the support observed in the XRD pattern after using the catalysts in the methanation reaction do not show significant variations.

The active metal, nickel, was found in its metallic form in both the reduced fresh and used catalysts. Nickel oxide was not detected in the reduced catalysts, nor in the used ones. Ni peaks were observed at 44.5° (111), 51.6° (200), and 76.7° (220) (Hu et al. 2019). The overlapping of some of the peaks of alumina and nickel produces their widening, which make it difficult the identification. Moreover, in the La-modified rGO-supported catalyst, nickel was combined with sulfur present in the graphene composition, forming a complex identified as Heazlewoodite (Hu et al. 2019).

The lanthanum detected in the XRD patterns was associated with La_2_(CO_3_)_2_(OH)_2_·H_2_O in the reduced rGO and LaCO_3_OH in the used rGO-supported catalyst while in the alumina-supported catalyst it was detected in a single La_2_O_2_CO_3_ phase. Furthermore, no diffraction peaks of La_2_O_3_ were detected in any XRD pattern, thereby suggesting that either this structure was amorphous or La_2_O_3_ was highly dispersed on the support (Cai et al. 2010). After the reaction step, the La_2_O_3_ dispersed into the 13Ni/14La-Al_2_O_3_ catalyst was transformed into the La_2_O_2_CO_3_ species. The addition of La to the Ni/Al_2_O_3_ freshly reduced catalyst produced the attenuation of the Ni reflection peaks, because of the interaction between La-Ni-alumina that increased the dispersion of the metal in the support (Hu et al. 2019; Liang et al. 2019; Garbarino et al. 2019). A similar effect was observed when comparing Ni_3_S_2_ and Ni, by adding La to the catalyst 13Ni/rGO, due to the strong interaction between Ni and La.

The XRD patterns of the catalysts supported in rGO showed several peaks that were not associated with the incorporated metals. These peaks represent the support, in the form of carbon (C) and a compound formed by carbon and silicon: silicon carbide or moissanite (SiC). The pattern observed after the reaction stage resembles that obtained for the rGO without the addition of metals to its composition. The peaks associated with CS_2_ that were observed for used catalysts, like carbonated lanthanum compounds, were not identified in the fresh catalysts, due to their formation during the reaction step. These components appeared by the deposition of carbon and sulfur on the metal centers and the support, during the reaction stage. Its presence might be the cause of the loss of activity of the catalyst and poisoning, which hinders the recovery of activity.

The value and the species identified were contrasted with the bibliography and collected in Table 3. In the case of the XRD pattern of rGO (not shown), two carbon species were identified with the codes JCPDS 026–1076 at 43° and 44.6° and 075–0444 at 25.9° and 44.6°.

**Table 3:** XRD peaks for the metallic species of the catalysts analyzed, determined graphically and contrasted with the bibliography

Species	JCPDS code	Value	Bibliography
Al_2_O_3_	077–0396	19.7, 34.0, 37.5, 44.5, 57.0, 61.0, 67.4, 85.0	(Paglia 2004; Segal et al. 2018)
C	075–0444 and 026–1076	25.9	(Hu et al. 2019)
Ni syn/Ni	004–0850/087–0712	44.5, 51.6, 76.7	(Hu et al. 2019)
NiAl_2_O_4_	073–0239	37.2, 44.2, 64.3	Hu et al. 2019
Ni_3_S_2_	076–1870	21.8, 31.2, 44.5, 50.0, 55.3, 73.4, 78.0	(Tian et al 2019)
La_2_(CO_3_)_2_(OH)_2_·H_2_O	046–0368	15.8, 20.5, 30.0, 30.3, 33.6, 36.7, 38.1, 43.9, 46.2, 48.8	(Kalai et al. 2017)
LaCO_3_OH	026–0815	17.8, 24.4, 30.3, 43.0, 43.9, 46.8, 50.0, 53.5, 57.2	(Lu et al. 2019)
La_2_O_2_CO_3_	025–0424	26.0, 30.5, 33.7, 44.6, 51.8	(Saravani and Khajehali 2016)

The mean Ni crystallite size was estimated by XRD from the Ni (111) reflexion at 2θ = 44.5° (Figs. 3 and 4), and results are listed in Table 2. Irrespective of the support and the presence of lanthanum, all catalysts were composed of small nickel crystallites with an average size of about 5–10 nm. After the reaction, an increase in crystallite size was observed in all the catalysts, which was a result of the sintering of metallic sites.

#### Temperature-programmed reduction (H_2_-TPR)

**Fig. 5:**
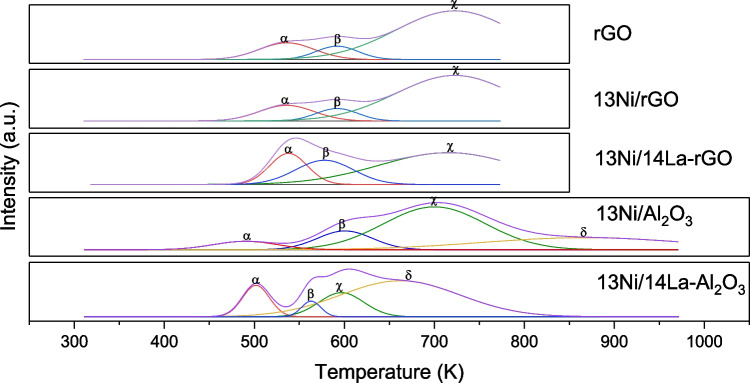
H_2_-TPR profiles of the investigated catalysts

Next, reducibility of the catalysts will be discussed. H_2_-TPR experiments were carried out not only to identify metallic phases present in each catalyst but also to analyze their interaction with the support. Figure 5 compares the reduction profiles of the prepared catalysts (along with a Gaussian-type peak deconvolution) while Table 4 lists the experimentally fitted contributions/values of these deconvoluted profiles. Starting with rGO-supported catalysts, three reduction peaks could be distinguished, centered at K, K, and K, depending on the nickel-support interaction. Thus, the former (373–523 K) belonged to amorphous NiO weakly interacting with rGO (α-type NiO), the peak at 523–773 K was attributed to nickel species with weak-medium interaction (β-type NiO), and the higher temperature peak was associated with the reduction of Ni^2+^ species mediumly interacting with the support (γ-type NiO). For alumina-supported catalysts, in addition to these three Ni species (α-, β-, and γ-NiO), another NiO phase, namely, δ-type NiO, was detected (located around 1023 K), which was related to the reduction of Ni^2+^ highly stabilized in a non-stoichiometric nickel aluminate structure (NiO-Al_2_O_3_). The stoichiometric nickel aluminate species (NiAl_2_O_4_) had a strong interaction with the support being harder to reduce needing a temperature higher than 1073 K. This species was not determined in the catalysts analyzed, under the evaluated operating conditions (Liang et al. 2019; Mo et al. 2019; Bizkarra et al. 2015; García-Lario et al. 2015; Lee et al. 2004).

**Table 4:** Gaussian fitting analysis of H_2_-TPR profiles of the catalysts studied

Catalyst	Peak area (a.u.)				Fraction of total area (%)			
	$\alpha$	$\beta$	$\gamma$	$\delta$	$\alpha$	$\beta$	$\gamma$	$\delta$
13Ni/Al_2_O_3_	6.78	28.57	13.2	3.55	13.01	54.84	25.34	6.81
13Ni/14La-Al_2_O_3_	7.53	9.61	38.25	2.67	12.97	16.55	65.88	4.60
rGO	15.25	9.27	74.14		15.46	9.40	75.15	
13Ni/rGO	32.66	19.84	158.78		15.46	9.39	75.15	
13Ni/14La-rGO	38.73	112.96	46.55		19.54	56.98	23.48	

The reduced graphene oxide, as support, had a base reducibility in the absence of any metal, due to the presence of oxygen functional groups that allowed the easy union of metal nanoparticles to its structure and it acted as anchoring or nucleation centers. The nickel that was incorporated into the structure was attached to these anchoring centers so that the peaks obtained in the H_2_-TPR pattern appeared at the same temperature, but being quantitatively higher (Hu et al. 2019). Specifically, the peak area determined in catalyst 13Ni/rGO was twice that obtained for rGO for peaks $\alpha$, $\beta$, and $\gamma$, due to the addition of the reducibility of metal, support, and their interaction.

The identification of a peak $\delta$ next to $\gamma$ in 13Ni/Al_2_O_3_ catalyst indicated a greater interaction between active metal and support (non-stoichiometric nickel aluminate formation). Despite the low amount of this species, its presence indicated a greater stability of the catalyst to deactivation by sintering but also a lower reducibility. In the 13Ni/Al_2_O_3_ catalyst, peak $\alpha$ shifted to a lower temperature, than in 13Ni/rGO, which indicated that the interaction between amorphous NiO and support was lower. On the other hand, the decrease in the area of peak α was balanced out by an increase in peak $\beta$.

TPR patterns of La-modified supports showed a splitting of the peaks at temperatures between 500 and 700 K. The $\alpha$ peak at 505 K was increased, corresponding with the rise of free NiO species in the alumina support, which was increased by adding La to the catalyst (Garbarino et al. 2019; Mo et al. 2019; Song et al. 2017). Based on the results and the literature observations, the incorporation of La weakened the interaction between NiO and Al_2_O_3_ support, by destroying partially metastable Ni–Al mixed oxide phase (Garbarino et al. 2019; Mo et al. 2019; Song et al. 2017). In this way, there was an increase in medium-strong interactions and the decrease in the reduction temperature of all the species present in the catalyst. The addition of lanthanum to the rGO support produced a similar effect to that observed for alumina, increasing the amount of medium–low interaction NiO ($\beta$ peak) with respect to that of medium-strong interaction NiO ($\gamma$ peak). In this sense, lanthanum caused the weakening of nickel interactions with the support for both alumina and rGO supports. A weaker interaction of the active metal with the support meant a greater reducibility of the catalyst, but it could increase the deactivation of the catalyst due to the breaking of the bonds of the active metal with the support, resulting in metal sintering.

Based on the results obtained and the literature, the catalysts activated by the reduction at 673 K must be almost completely reduced for the reaction conditions between 498 and 773 K for CO_2_ methanation.

#### HR-STEM

**Fig. 6:**
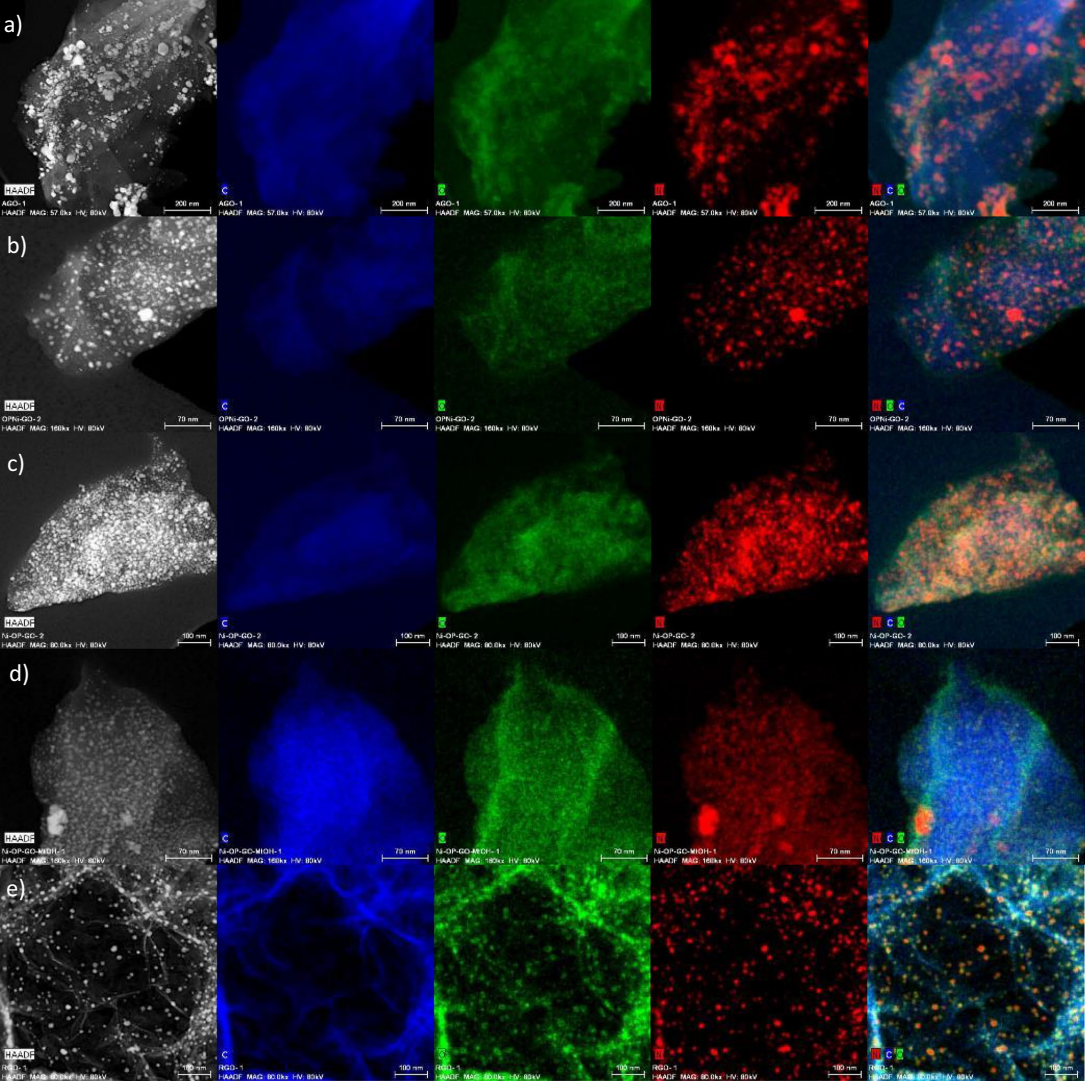
STEM-EDX images and element mapping of **a** 13Ni/AGO, **b** 13Ni/BGO, **c** 13Ni-Ol/GO, **d** 13Ni/Ol-GO, **e** 13Ni/Ol-GO Met, and **f** 13Ni/rGO freshly reduced catalyst

**Fig. 7:**
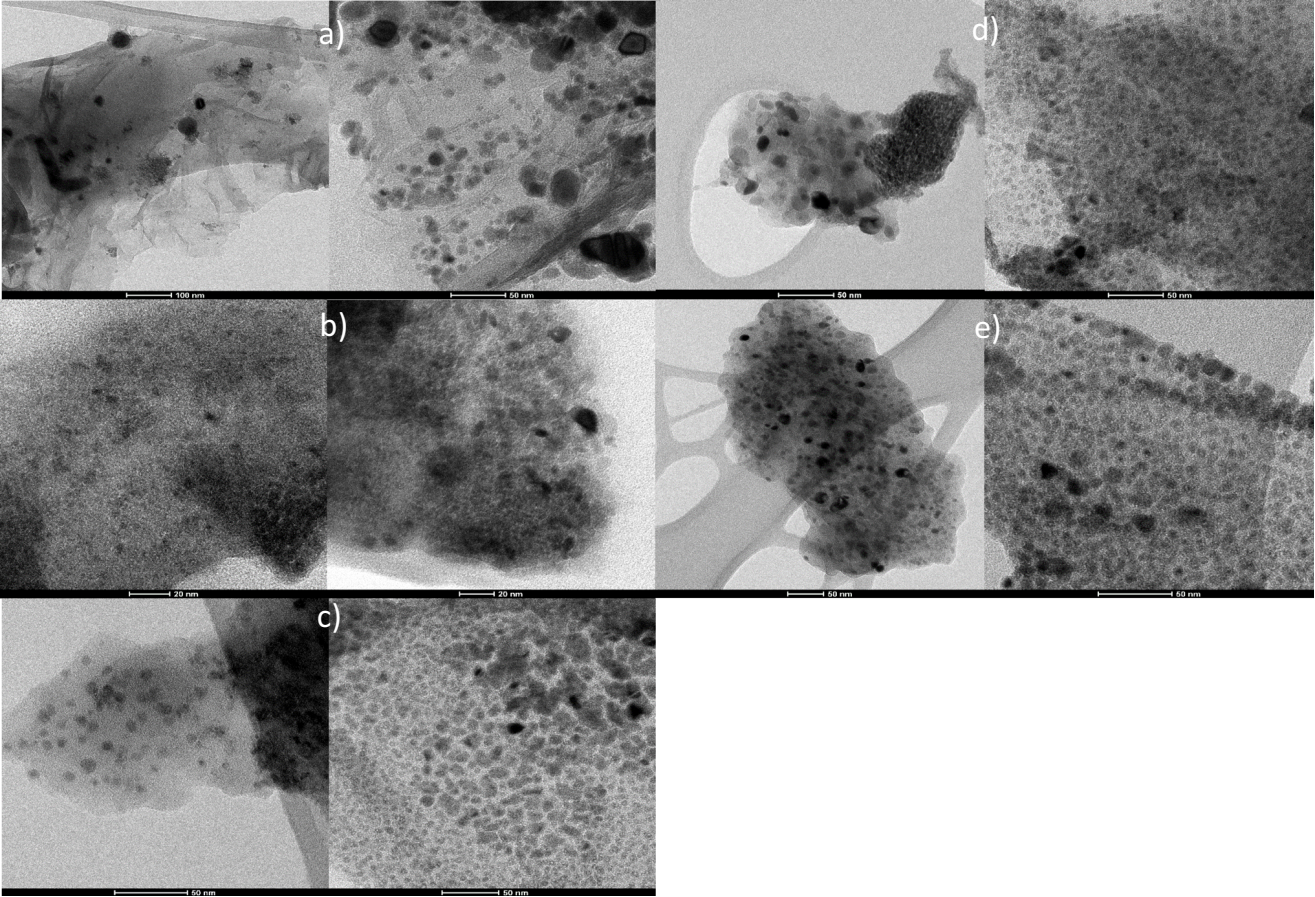
STEM-EDX images and element mapping of **a** 13Ni/AGO, **b** 13Ni-Ol/GO, **c** 13Ni/Ol-GO, **d** 13Ni/Ol-GO Met, and **e** 13Ni/rGO used catalyst

STEM combined with EDX was employed to analyze the morphology of the catalysts, in addition to mapping its surface to locate and identify the content of C, O, N, S, and Ni elements. Figure 6 shows the STEM-EDX images; mapping results were also depicted for the following catalysts: 13Ni/AGO, 13Ni/BGO, 13Ni-Ol/GO, 13Ni/Ol-GO, 13Ni/Ol-GO Met, 13Ni/rGO, and 13Ni/14La-rGO. GO-based catalysts (13Ni-Ol/GO, 13Ni/Ol-GO, and 13Ni/Ol-GO Met) showed the presence of sulfur dispersed on the catalyst surface, as part of the GO support composition due to its synthesis method. In the 13Ni-Ol/GO catalyst, the amount of sulfur observed was considerably lower than for the other two catalysts, probably due to the elimination of the oleylamine impregnation step, which in turn allowed a stronger anchoring of sulfur species on the support. These undesirable species were partially removed when the metal was simultaneously impregnated with the oleylamine through a single stage. However, based on STEM images, in the absence of sulfur less nickel was fixed. This metal was well dispersed in all the catalysts, mainly due to the formation of Ni-C σ bonds that promoted the stacking of the layers (Hu et al. 2019). Thus, the main difference observed among the graphene-based catalysts was the particle size that depends mainly on the support. The Ni particle sizes determined for the different catalysts are listed in Table 4, for both freshly reduced and used catalysts. With the exception of the 13Ni/AGO catalyst, whose Ni particle size was 68.5 nm, the other catalysts showed small particle size values, distributed homogeneously throughout the surface of the support. As inferred from Table 5, the addition of lanthanum led to slightly bigger nickel particles (5–20 nm), maintaining a homogeneous dispersion (Garbarino et al. 2019). It should be pointed out that particle sizes determined by HR-STEM (Fig. 7) analysis were comparable to crystallite sizes estimated by XRD, with the exception of 13Ni/14La-rGO used catalyst, which due to sintering caused by H_2_S and temperature increases its size significantly, measuring the Ni_3_S_2_ crystallite size. When compared with XRD data, it was reasonable to assume that on average active nickel particles (around 5–25 nm) were composed of one or at most two–three crystallites (about 5–10 nm), thereby proving the mainly monocrystalline nature of the samples.

**Table 5:** Metallic particle size of the different catalysts determined by STEM and XRD

	Particle size by STEM (nm)		Particle size by XRD (nm)	
	Fresh catalyst	Used catalyst	Fresh catalyst	Used catalyst
13Ni/AGO	$69\pm 18$	$23\pm 7$		
13Ni/BGO	$12\pm 5$	–		
13Ni-Ol/GO	$23\pm 6$	$7\pm 2$		
13Ni/Ol-GO	$26\pm 6$	$14\pm 3$		
13Ni/Ol-GO (Met)	$8\pm 2$	$6\pm 2$		
13Ni/alumina	$10\pm 3$	$12\pm 3$	~ 5	~ 10
13Ni/14La-alumina			~ 5	~ 15
13Ni/rGO	$11\pm 4$	$13\pm 4$	~ 6	~ 8
13Ni/14La-rGO			~ 7	~ 80*

*Average of Ni_3_S_2_ crystallite size (at 22°) instead of Ni (44.5°)

**Fig. 8:**
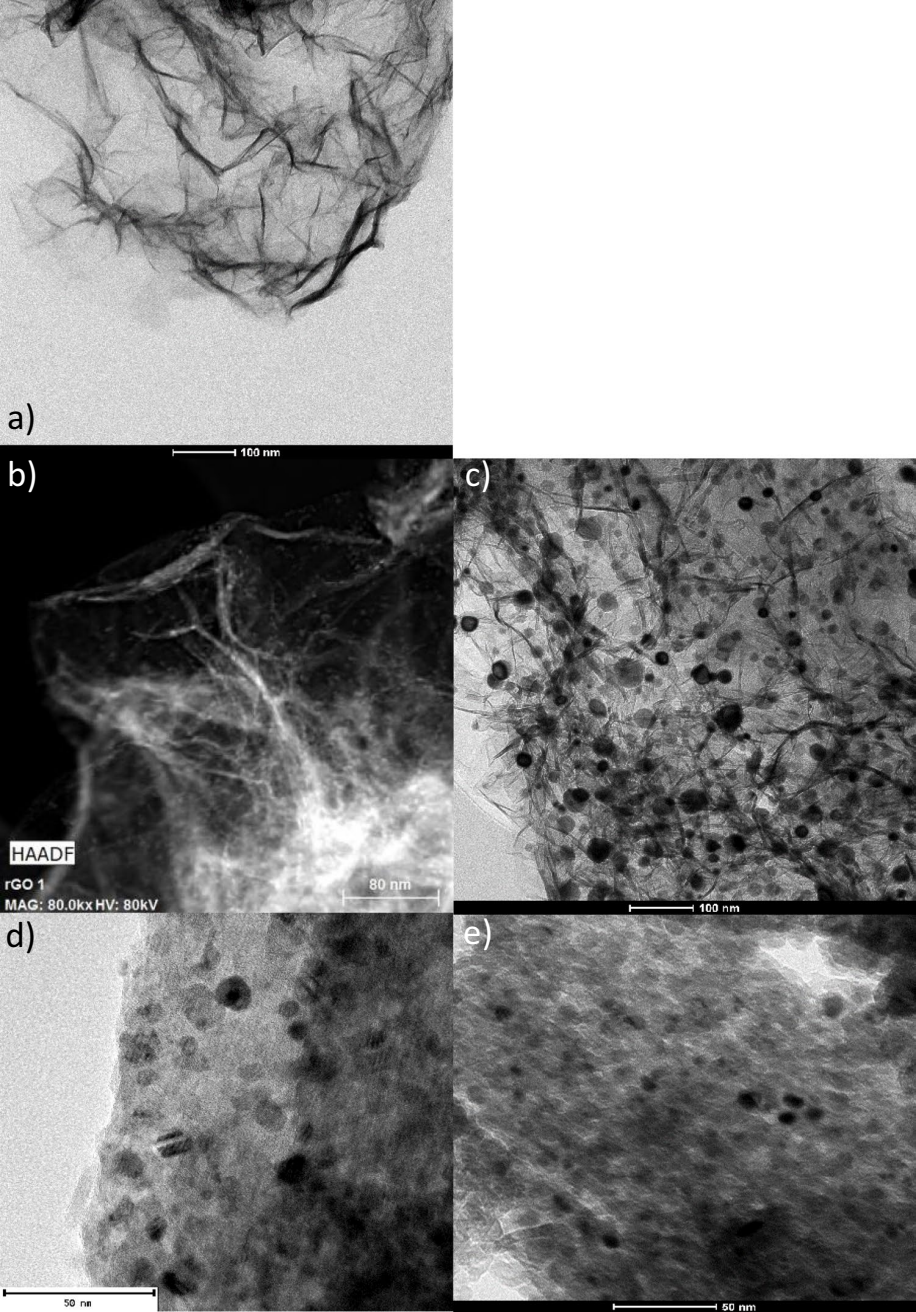
STEM micrographs of **a** rGO, **b** 13Ni/rGO fresh, **c** 13Ni/rGO used, **d** 13Ni/Al_2_O_3_ fresh, **e** 13Ni/Al_2_O_3_ used, **f** 13Ni/14La-Al_2_O_3_ fresh, **g** 13Ni/14La-Al_2_O_3_ used, **h** 13Ni/14La-rGO fresh, and **i** 13Ni/14La-rGO catalyst

After reaction, used catalysts were also studied by HR-STEM and the corresponding images are showed in Fig. 8. This analysis revealed no significant morphological differences when compared to freshly reduced catalysts. However, in terms of particle size, some variations were detected. For catalysts 13Ni/Al_2_O_3_, 13Ni/14La-Al_2_O_3_, 13Ni/rGO, and 13Ni/14La-rGO, a growth of their size was identified (from 10.0 to 13 nm), which was mainly attributed to the sintering caused by high temperatures and the action of H_2_S in the catalysts (Yuan et al. 2015). Conversely, the particle size of graphene oxide-based catalysts 13Ni/AGO, 13Ni-Ol/GO, 13Ni/Ol-GO, and 13Ni/Ol-GO (Met) was reduced owing to the loss of the oxygen that made up the Ni complexes. Therefore, it could be stated that in the absence of this oxygen anchoring the metal to the support with strong interaction, metallic particles were divided into smaller aggregates (Hu et al. 2019).

#### XPS

**Fig. 9:**
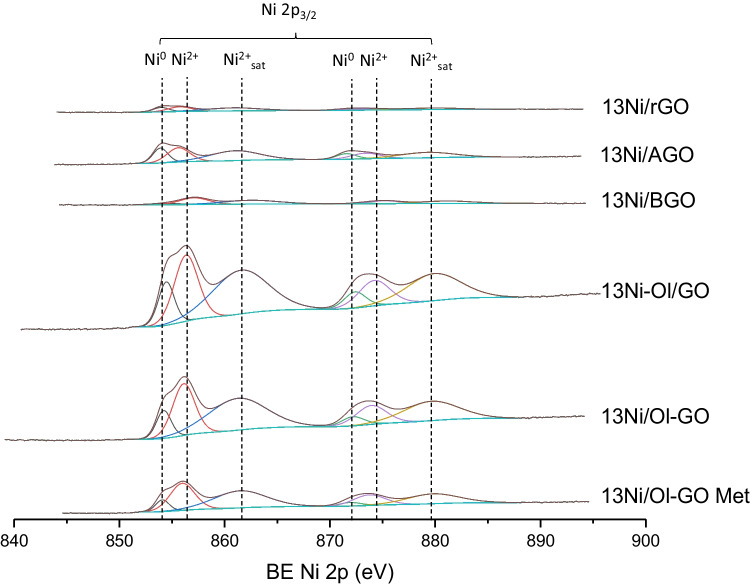
XPS spectra of the Ni 2p_3/2_ region of fresh Ni catalysts based on graphene

**Fig. 10:**
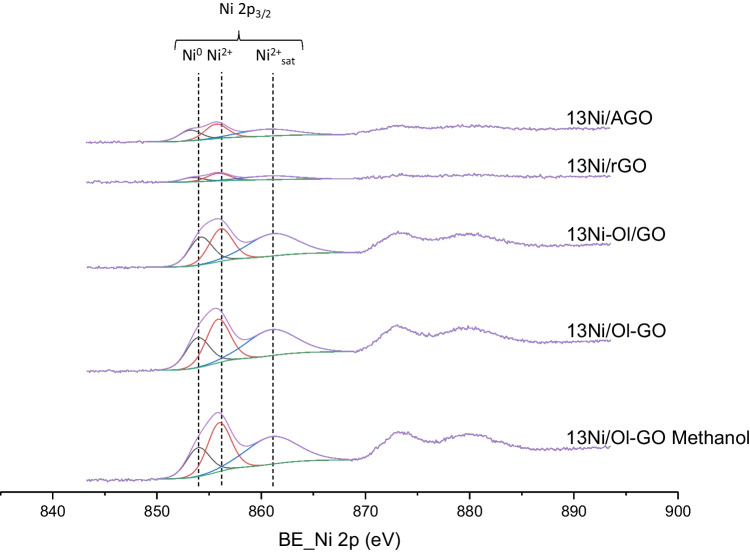
XPS spectra of the Ni 2p_3/2_ region of used Ni catalysts based on graphene

X-ray photoelectron spectroscopy was used to provide information about the oxidation state and the chemical environment of the nickel present on the surface of all the investigated catalysts. The Ni 2p_3/2_ region of each freshly reduced sample is plotted in Figs. 9 and 10 while the spectra of the used catalysts are depicted in Figs. 10 and 12. In both cases and according to literature, Ni 2p_3/2_ spectra exhibited a doublet of two multiple splits located in the range of 854–862.6 eV and 865–890 eV (NIST XPS DATABASE 2019). Focusing on the lower energy multiple, three nickel contributions were detected at 854–854.5, 855.7–857.2, and 860.8–862.6 eV, which were attributed to Ni^0^, Ni^2+^, and Ni^2+^ satellite, respectively (Gnanakumar et al. 2018).

As for alumina-supported catalysts, the contribution of metallic nickel was higher than the nickel oxide phase, thanks to the in situ reduction stage carried out in the XPS chamber. However, this reduction treatment was not applied to graphene-supported catalysts, and as a result, instead of metallic Ni, Ni^2+^ was the main oxidation state on the surface of these catalysts. At this point, it should be mentioned that is a very common issue the oxidation of metallic particles during the sample preparation and introduction into the XPS chamber. Nevertheless, when compared to the used counterparts, the intensity of superficial NiO was lower, suggesting that after reaction metallic nickel was partially oxidized (Abdel-wahab et al. 2016). This treatment was not applied in graphene-supported catalysts, with a greater intensity of nickel oxide peak, than of metallic nickel. However, despite observing a greater intensity in the nickel oxide peak, it was lower than that obtained in the case of the used samples, where the intensity of the oxide peak was much greater than that of the metal.

**Fig. 11:**
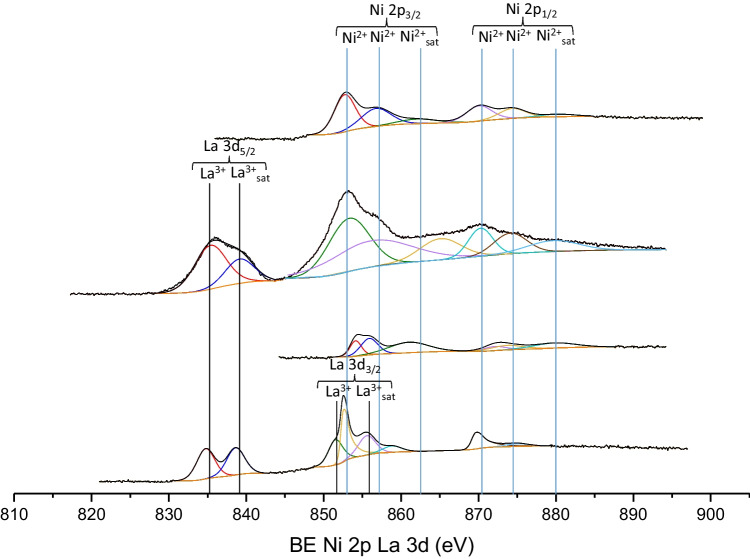
XPS spectra of La 3d_5/2_, La 3d_3/2_, Ni 2p_3/2_, and Ni 2p_1/2_ regions of fresh catalysts

**Fig. 12:**
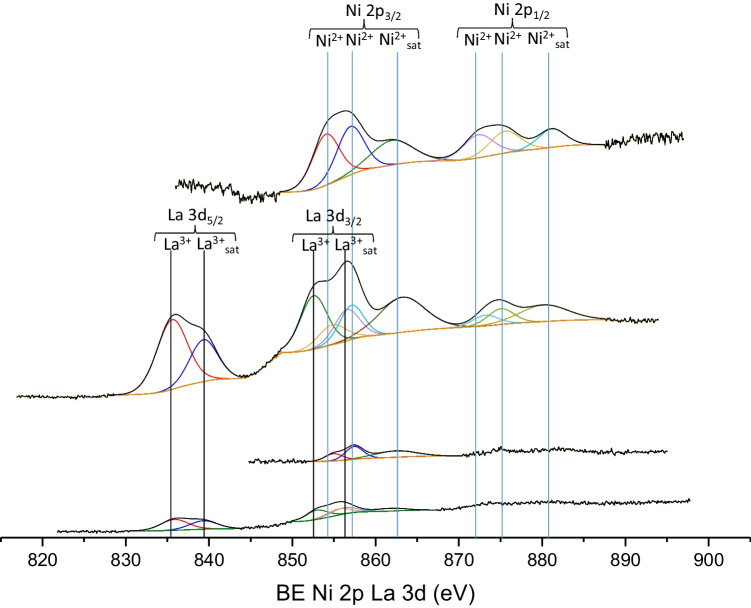
XPS spectra of La 3d_5/2_, La 3d_3/2_, Ni 2p_3/2_, and Ni 2p_1/2_ regions of used catalysts

XPS of the La 3d_5/2_ and La 3d_3/2_ core level spectra of the 13Ni/14La-Al_2_O_3_ and 13Ni/14La-rGO samples (Figs. 11 and 12) clearly evidenced the presence of this promoter on their surface. Indeed, the position of the main peak at 835.0 eV and the characteristic distance between this main peak and its satellite of about 4 eV undoubtedly confirmed the existence of La_2_O_3_ on the surface of the catalysts (Garbarino et al. 2019). However, it was not possible to quantify the Ni interacting with La due to the superposition with the Auger LMM line of Ni 2p 3/2 and La 3d 3/2 in the case of the catalyst 13Ni/14La-Al_2_O_3_ and 13Ni/14La-rGO. Nonetheless, the Ni 2p_3/2_ corresponding triplet moves toward lower binding energy, while the La 3d_3/2_ doublet increases its bond energy, producing the overlapping of the corresponding peaks, confirming the interaction that occurs between La^3+^ and Ni^2+^ (Garbarino et al. 2019).

In addition to the identification of all elements present at the surface level, this technique was also useful for the quantification of these species, namely, metallic nickel, carbon, oxygen, sulfur, and lanthanum modifier, and for the determination of their interactions. In the case of catalysts supported on graphene, this technique allows to identify the amount of active metal (Ni), carbon and oxygen, and nitrogen, sulfur and support modifier (La) when present and, through the oxidation state, determine the bonds in which they were found. The results of this analysis for both freshly reduced and spend catalysts are summarized in Table 6. As can be deduced, the proportion of Ni varies depending on the support used. In inorganic-supported catalysts, the nickel content of the surface was lower than that for organic-supported catalysts, based on graphene. Graphene was made up of extensive but shallow layers, so most of the metal content incorporated in these supports can be detected on the surface by XPS. When compared to ICP results the tendency of Ni content varied, thus suggesting that the composition of the bulk was different from the surface.

**Table 6:** Surface atomic values obtained by XPS analysis for the investigated catalysts

	Fresh catalyst						Used catalyst					
	C/Al (%)	O (%)	Ni (%)	N (%)	S (%)	La (%)	C/Al (%)	O (%)	Ni (%)	N (%)	S (%)	La (%)
13Ni/AGO	73.8	15.4	6.9	3.9	–	–	85.4	9.8	2.3	2.4	–	–
13Ni/BGO	16.5	32.9	2.1	1.4	0.3	–	–	–	–	–	–	–
13Ni-Ol/GO	44.3	34.3	13.2	5.0	3.3	–	52.2	29.4	6.4	2.9	–	–
13Ni/Ol-GO	16.7	52.8	21.1	2.6	6.8	–	65.5	22.5	7.2	3.3	1.5	–
13Ni/Ol-GO (Met)	22.1	50.4	16.5	3.7	7.3	–	57.9	28.8	8.1	3.5	1.7	–
13Ni/Al_2_O_3_	40.4	56.5	2.7	–	–	–	37.7	59.1	2.6	–	–	–
13Ni/14La-Al_2_O_3_	35.7	60.3	*	–	–	3.4	26.7	64.9	5.3	–	–	3.1
13Ni/rGO	88.3	9.8	1.1	0.8	–	–	91.6	6.0	0.5	–	–	–
13Ni/14La-rGO	86.9	9.4	2.0	–	–	1.7	83.4	14.7	0.9*	–	–	1.0

*Presence of Ni, but cannot be quantified (2p line of Ni overlapped with 3d line of La)

According to the results obtained by ICP and HR-STEM-EDX, values of XPS also indicated that, except for 13Ni/BGO catalyst, nickel was mostly contained into the bulk of the catalysts or linked to carbon atoms in deep layers of the catalysts. On the other hand, sulfur was only detected in the freshly reduced catalyst supported on graphene oxide, denoting that it was not eliminated in the synthesis step. This content was higher for oleylamine-incorporated catalysts, as verified by HR-STEM. As for used samples, the amount of this sulfur decreased, even disappearing despite feeding H_2_S, due to subsequent reaction and regeneration steps. These steps may remove the sulfur content from the catalyst surface, not being detected with this technique. As general trend, the amount of carbon in graphene-based catalysts increased significantly with respect to fresh catalysts, which in turn caused a reduction of oxygen, nitrogen, and nickel proportions. This fact may be due to the appearance of carbon deposits from the reaction, which covered the catalyst surface and reduced its catalytic activity. The amount of nitrogen detected by the XPS analysis depends on the catalyst preparation technique used and of the amine groups incorporated so that the highest content was observed for the 13Ni-Ol/GO catalyst when nitrogen was incorporated with oleylamine. In the other catalysts that incorporate oleylamine, the nitrogen content was lower and probably lost after two stages of calcination, as shown in the CHN analyses in Table 7. In the 13Ni/AGO catalyst, the high nitrogen content was due to the group amino incorporated in its preparation. The rest of the catalysts have a lower nitrogen content, corresponding to the amount originally present in GO or rGO originally.

**Table 7:** Carbon, hydrogen, and nitrogen content of investigated catalysts determined/measured by CHN

	C (%)	H (%)	N (%)
13Ni/AGO	65.8	2.5	3.9
13Ni/BGO	4.9	0.4	0.4
13Ni-Ol/GO	43.6	1.5	3.9
13Ni/Ol-GO	6.2	1.2	0.8
13Ni/Ol-GO (Met)	20.6	1.7	1.8
13Ni/rGO	68.2	–	1.25

Finally, the higher oxygen content than carbon detected in XPS for catalysts incorporating oleylamine can be explained by the greater presence of oxygenated hydrocarbons, such as the hydrocarbon chain that make up oleylamine. Therefore, as the amount of oleylamine increases, so does oxygen (as verified in elementary CHN analyses). In turn, the higher content of functional oxygen groups, the anchoring of nickel is facilitated.

### Activity tests

**Fig. 13:**
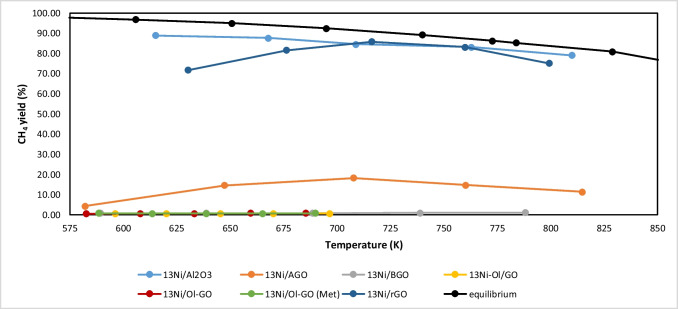
Methane yield obtained for investigated supported nickel catalysts

The catalytic behavior of synthesized catalysts, based on 13 wt.% Ni supported on graphene-based oxides or alumina and modified with 14 wt.% lanthanum, was investigated for CO_2_ methanation. Runs were carried out with 200 mg of catalyst, at temperatures between 573 and 773 K and a weight hourly space velocity of 38.3 h^−1^. For the evaluation of the catalytic performance, methane yield parameter was selected (Eq. 4), and the obtained results are reported in Fig. 13.

Firstly, the very low catalytic performance of oleylamine-containing aminated catalysts (13Ni-Ol/GO, 13Ni/Ol-GO, and 13Ni/Ol-GO (Met)) should be highlighted, since these samples showed methane yields close or equal to 0.

In the case of 13Ni/BGO catalyst, the observed activity was also negligible. Indeed, owing to its synthesis method, the metal content of this catalyst in the bulk (1.3 wt.%) as well as on the surface (2.1%) was very low, and as a result, despite its good dispersion (as observed by STEM), it was not enough to increase the methane yield above 3%. On the other hand, the catalytic activity of the 13Ni/AGO catalyst was significantly better than its counterpart 13Ni/BGO, reaching a maximum methane yield of 18.3% at 708 K. This improved activity, when compared to oleylamine-containing catalysts, was mainly attributed to the fact that having 12 carbon atoms instead of 18 of the oleylamine facilitated the access of the reagents to the catalyst. Even so, this catalyst was less active than catalysts supported on rGO and alumina, which achieved the highest methane yield (around 90% at 710 K). Specifically, both 13Ni/Al_2_O_3_ and 13Ni/rGO samples exhibited a great catalytic performance, being practically equivalent at intermediate temperatures (from 700 to 760 K) although both at lower temperatures (600–700 K) and at high thermal levels (> 760 K) the 13Ni/Al_2_O_3_ catalyst was superior to 13Ni/rGO.

**Fig. 14:**
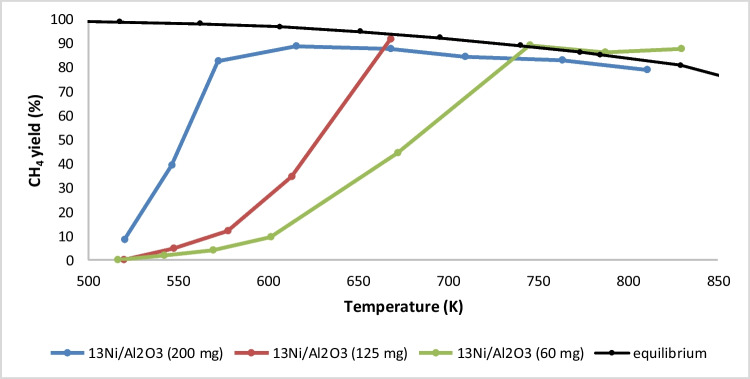
Comparison of methane yield for different quantities of 13Ni/alumina catalyst

Based on these results, the promising 13Ni/Al_2_O_3_ and 13Ni/rGO catalysts were the samples selected to be optimized by modifying their supports with lanthanum. Nevertheless, prior to this study, a complementary analysis was carried out over 13Ni/Al_2_O_3_ sample in order to determine the optimum catalyst amount. Thus, the catalytic activity of CO_2_ methanation reaction was evaluated from 525 to 850 K using three different amounts of 13Ni/Al_2_O_3_, namely, 200 mg (38.3 h^−1^), 125 mg (60.8 h^−1^), and 60 mg (128.75 h^−1^), and the obtained results are shown in Fig. 14.

As expected, as the amount of catalyst was reduced or the WHSV was increased, the temperature needed to reach the maximum yield set by the thermodynamic equilibrium increased. With a load of 200 mg of catalyst in the reactor, the maximum conversion was reached at 572 K (close to equilibrium), while for 125 mg the temperature needed to be raised to 668 K and for an amount of 60 mg the equilibrium temperature was not reached until 745 K. For a catalyst quantity of 60 mg, equilibrium was reached in the study temperature range, with a little more difficulty to increase performance.

**Fig. 15:**
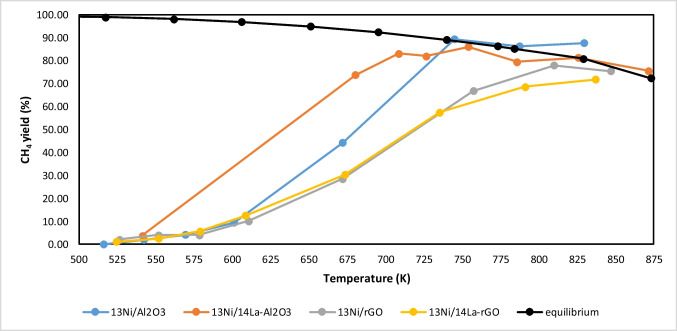
Methane yield obtained for 60 mg of catalysts supported on alumina and rGO

Once the quantity of 60 mg of catalyst was chosen, the effect of lanthanum (14 wt.%) on the activity of both alumina and rGO-supported catalysts was examined. For that purpose, runs were performed operating with a WHSV of 128.75 h^−1^ and in the temperature range of 598–773 K. The corresponding activity results are depicted in Fig. 15.

The 13Ni/Al_2_O_3_ catalyst achieved a maximum methane yield of 90% at 745 K, as already seen at Fig. 15, while its analogous La-modified sample (13Ni/La-Al2O3) reached the same value at lower temperatures (and 89% at 727 K). That means that the incorporation of La into alumina support considerably reduced the temperature necessary to reach equilibrium. However, for rGO-supported catalysts, no significant improvement was observed when La was incorporated, being the light-off curve of both catalysts (as-synthesized and modified) practically identical up to 725 K and only slightly higher for La-modified catalyst (13Ni/La-rGO) from 725 to 798 K. Furthermore, and despite the modification with La, the alumina-supported catalyst was still more active than the analogous rGO-supported catalyst. This fact denoted that the method of incorporating Ni on a support with larger surface area, such as the rGO, and its subsequent modification with lanthanum (La-rGO) was not successful for achieving greater dispersion of the active centers on the surface of the support and therefore neither for improving the activity of rGO-supported catalysts with respect to alumina-supported samples.

**Fig. 16:**
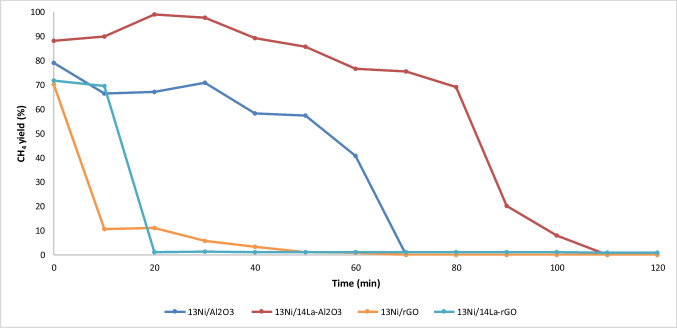
Catalyst deactivation due to 50 ppm of H_2_S at 773 K

As a final research point, the resistance of these for catalysts against H_2_S was examined, since this compound constitutes one of the most common poisoning factors in CO_2_ methanation. For this purpose, the last four (13Ni/Al_2_O_3_, 13Ni/La-Al_2_O_3_, 13Ni/rGO, and 13Ni/La-rGO) catalysts were tested at 773 K, the highest performance temperature, in the presence of 50 ppm of H_2_S, which was co-fed with the other reaction gases, and the time necessary for the complete deactivation of the catalysts was determined. The methane yield evolution with time on stream for each catalyst is plotted in Fig. 16. The presence of H_2_S in the reaction stream rapidly reduced the activity of rGO-supported catalysts, deactivating them completely in only 20 min in the case of the 13Ni/14La-rGO and in 50 min in the case of the 13Ni/rGO, although the latter suffered a vertiginous decrease to 10% yield in just 15 min. This rapid deactivation was expected considering the high operation temperature, which reduced the catalytic performance with time on stream by thermal deactivation. Regarding alumina-supported catalyst, the addition of 14 wt.% of La to 13Ni/Al_2_O_3_, apart from improving the obtained methane yield, enhanced markedly its resistance to H_2_S poisoning, lengthening the deactivation time from 70 min for the 13Ni/Al_2_O_3_ to 110 min for the catalyst 13Ni/14La-Al_2_O_3_.

Immediately after the complete deactivation of the catalysts, H_2_S was eliminated from the feed, and again, the methane yield of the catalysts was analyzed. However, in all cases, catalysts did not recover the activity.

Finally, in order to complete the research done in this work, the possibility of catalyst regeneration was examined. To this end, deactivated samples were treated with a mixture of 3% O_2_ and N_2_ at 773 K, in order to eliminate either the sulfur or coke deposits, that is, the main responsible for the decline in activity. After calcination, the catalysts were reactivated by reduction with H_2_ (following the conditions aforementioned in the experimental section) and their activity was again evaluated by means of methane yield at 773 K. From the obtained results (not shown), it was clearly inferred that after regeneration treatment catalysts were not able to recover their previous methane yield, showing values of 0%. This finding was probably connected to the fact that during the reaction and/or deactivation stage CO could have appeared as a by-product of the reverse water gas shift reaction or from the partial oxidation of the coke in the regeneration step. Nonetheless, the presence of this gas has not been detected.

## Discussion

In this work, graphene in the form of aminated graphene oxide (AGO) and reduced graphene oxide (rGO) has been used as support. In addition, other graphene derivatives have been compared in the methanation reaction to avoid the hot spots that may be produced by the exothermicity of the reaction, employing the heat conductive capacity showed by graphene and its derivatives. Among the catalysts prepared, the best support for nickel as an active metal was rGO. This catalyst was in turn doped with lanthanum, showing very small differences in its activity and in both cases being lower than catalysts with the same active metal supported in alumina, with which it was compared.

The use of nickel-based catalysts as active metal presents in different activity values depending on the type of support used. The metal-support interaction was a factor of great influence on the activity of a catalyst. That is why, catalysts based on GO modified with oleylamine (13Ni-Ol/GO, 13Ni/Ol-GO, and 13Ni/Ol-GO (Met)) presented such low activity values. The addition of this aminated group was proposed to offer a hydrophobic environment that facilitated the exit of water as a by-product, facilitating the methane formation reaction. The union of the oleylamine and the active nickel centers was produced homogeneously by the surface of GO and there was interaction between them, as determined by STEM characterization. However, probably this interaction hindered the adsorption of reactants on the metal sites.

Something similar occurred in catalyst supported on GO reduced with MeAB (13 Ni/BGO). In this case, MeAB was incorporated into the catalyst, reducing the proportion of metal centers. It was observed that the proportion of nickel with respect to the total decreased considerably from 13% to 1.5%. Despite having a lower nickel content than 13Ni/Ol-GO, 13Ni/Ol-GO (Met), and 13Ni-Ol/GO, the performance is higher (3%). Considering the moles of CH_4_ per minute and mole of nickel, it is observed that the efficiency of 13Ni/BGO (1.63) catalyst is greater than that of 13Ni/Ol-GO (0.01), 13Ni/Ol-GO (Met) (0.02), 13Ni-Ol/GO (0.03), and 13Ni/AGO (0.70) catalysts. However, it does not reach the value of the 13Ni/Al_2_O_3_ (4.55), 13Ni/14La-Al_2_O_3_ (3.93), 13Ni/rGO (4.03), and 13Ni/14La-rGO (3.61) catalysts.

On the other hand, the catalyst based on graphene oxide aminated with oleylamine (13Ni/AGO) showed an agglomeration of nickel particles sites, distributed evenly across the surface of the support. These centers agglomerated together with functional nitrogen and oxygenated groups that served as nucleation or anchoring centers for the active metal, thus facilitating and strengthening the metal-support interaction, as observed by HR-STEM. However, due to a lower dispersion of active centers, the catalytic activity achieved was lower than expected for the amount of nickel present in this catalyst.

**Fig. 17:**
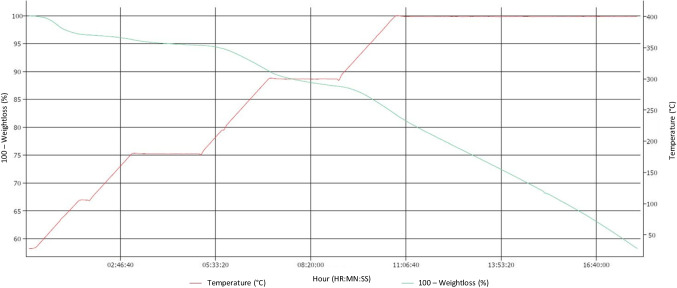
Loss of mass of the support rGO with the temperature, with increase of 1 K/min up to 673 K, keeping at constant temperature until the total loss of mass

In 13Ni/rGO, the support was partially reduced, but unlike the 13Ni/BGO catalyst, it retains oxygen atoms tightly bound to the GO structure by forming functional groups of oxygen and eliminating the most weakly bound oxygen atoms. These oxygen functional groups facilitated the deposition, anchoring, and dispersion of the Ni metal particles on the rGO structure homogeneously, as observed in STEM. The bond that occurs between metal and support was a strong bond, as confirmed by TPR analysis, resulted in high stability of the catalyst at temperature and therefore high activity of the catalyst in the reaction. The ability to conduct heat quickly through its surface reduced of hot spot formation usually present in the exothermic methanation reaction resulted in better temperature control that reduces catalyst deactivation. However, the bonds between the oxygen functional groups and the organic structure of the support have greater sensitivity to temperature, which causes a progressive deactivation of the catalyst as the temperature increases. This was further confirmed with TGA (Fig. 17), where the progressive loss of support mass can be observed as the temperature increases in an inert atmosphere. It was significant that the loss of mass occurs despite the increase in temperature with a small ramp and that it continues to decrease by keeping the temperature constant at a value of 673 K.

The rGO catalyst exhibits greater activity than the rest of graphene-based catalysts, but due to its temperature sensitivity, it is necessary to improve the activity in the methanation reaction, as it could only be used at moderate temperatures to increase the life of the catalyst and, thus, with a lower value of WSHV to improve performance at these conditions.

**Fig. 18:**
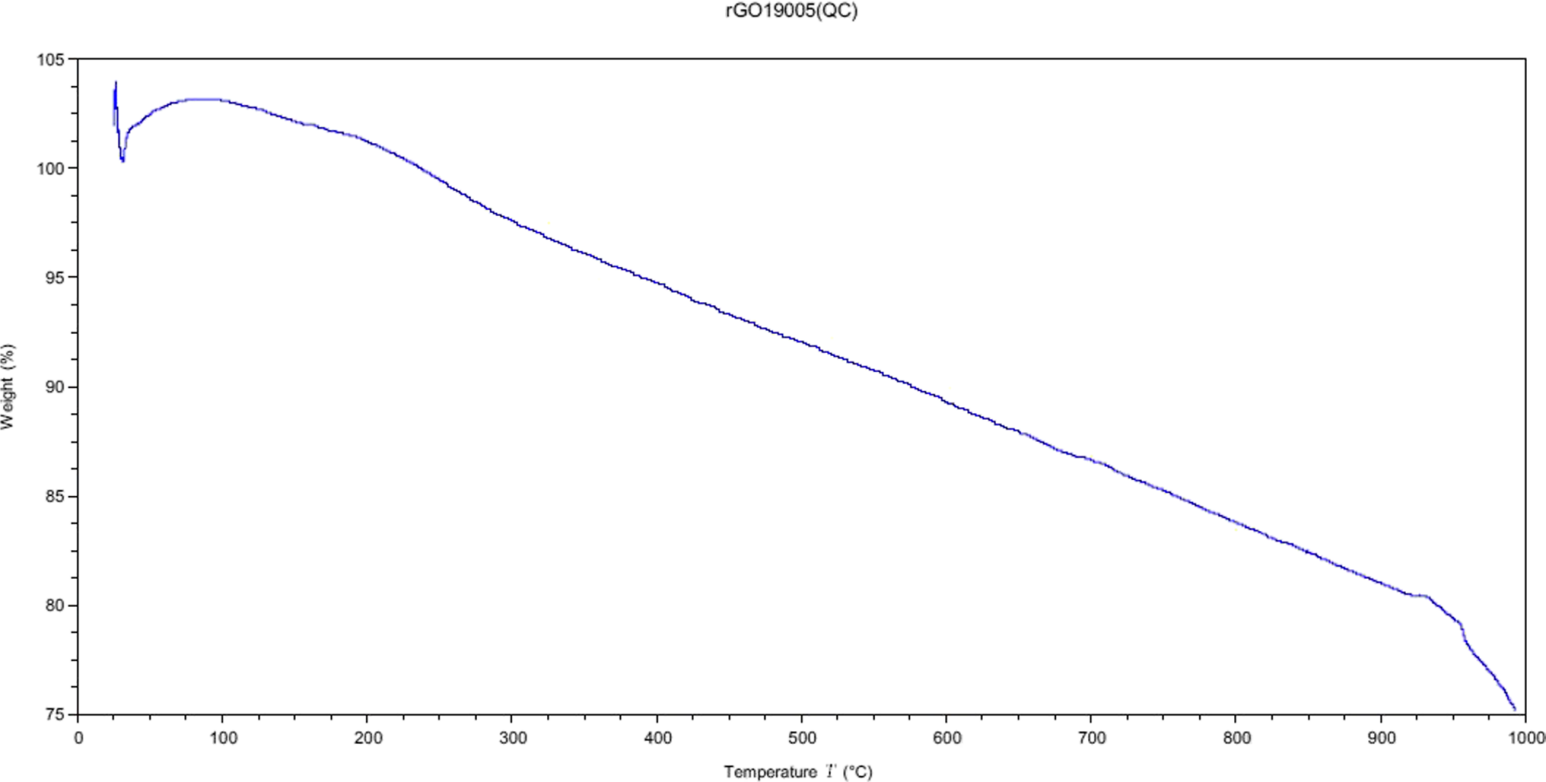
Loss of mass of the support rGO with the temperature, with increase of 10 K/min up to 1273 K (Graphenea)

An inorganic support, such as that of the alumina, guarantees greater thermal stability against deactivation of the catalyst at studied temperatures (498–773 K), compared to graphene oxide-derived catalysts. In fact, the 13Ni/Al_2_O_3_ catalyst achieves higher methane yield (89.5%) at a lower temperature (745 K) than the 13Ni/rGO catalyst (78% at 810 K), making it a more active catalyst, with a greater number of metal centers and more accessible. This contrasts with the results obtained in the work of Hu et al. (2019), in which they incorporate 20 wt.% nickel by wet impregnation on rGO, having reduced graphene via hydrazine hydrate. The authors determined that nickel-supported catalysts in rGO achieved higher methane yield (80%) at a lower temperature (623 K) than alumina-supported catalysts (65%). In addition, they demonstrated that their 20Ni/rGO catalyst maintained its activity over time at a temperature of 723 K, which justifies its stability against thermal deactivation. The difference in the results with catalyst 13Ni/Al_2_O_3_ was mainly due to the operating pressure, being the atmospheric in the work of Hu et al. (2019). The 13Ni/rGO catalyst, despite the differences in operating pressure, maintains a similar methane yield value, at temperature of 810 K. The thermal stability of rGO, like that achieved in the work of Hu et al. was achieved with an increase in the ramp to raise the temperature of the system, from 1 K/min to 10 K/min. With these new conditions, 1273 K was reached, without losing more than 20% of mass, as shown in Fig. 18 in TGA.

Methanation has been studied extensively using nickel catalysts supported on alumina. However, the vast majority of these works were carried out under atmospheric pressure conditions. In Italiano et al.’s (2020) work, a methane yield of 75% was reached at 673 K with 15Ni/Al_2_O_3_. In Quindimil et al. (2019), 80% was reached at 673 K with 12Ni/ Al_2_O_3_. In Garbarino et al. (2019), they reached up to 85% at 673 K with 16.7Ni/Al_2_O_3_. In the research carried out by Ahmad et al. (2017), 87% was obtained at 573 K with 12 Ni/Al_2_O_3_, which was close to 89.5% at 672 K that was obtained for 13Ni/Al_2_O_3_, when working with 10 bar of pressure and 128.75 h^−1^ of WSHV.

The addition of lanthanum, as support modifier, improves the catalytic activity of nickel in the temperature range between 523 and 750 K. The work of Garbarino et al. (2019) shows a result very similar to that observed in this work by adding lanthanum as a promoter, improving the yield to methane from 82% to 623 K for 16.7Ni/Al_2_O_3_, up to 92% at 623 K for 16.7Ni-14La/Al_2_O_3_ under atmospheric pressure. The addition of lanthanum to the nickel catalyst improves the catalytic activity, as an effect of a greater interaction between nickel-lanthanum-alumina. This was justified by the interaction observed in XRD and H_2_-TPR, whereby there was a weakening of the signal corresponding to the nickel peaks, especially that corresponding to the nickel centers more strongly attached to the support. However, there was also a slight decrease of the peaks corresponding to the alumina, which indicates that there was also an interaction of the lanthanum with the support. XPS analysis confirms the existence of an interaction between La and Ni, determined by the superposition of its spectra. This interaction results in the reduction of the nickel particle size and an improvement in the dispersion of the metal through the surface of the support. This effect was more significant in the catalyst supported on alumina than in the one supported on rGO, due to the above mentioned interaction between lanthanum and alumina. Due to the interaction between La, Ni, and alumina, the resistance of the catalyst against H_2_S poisoning was increased, making more difficult to block the active centers of the metal. This effect was mainly the cause of the contribution of alumina, since as observed in Fig. 16 the presence of lanthanum in the 13Ni/14La-rGO catalyst did not mean an improvement in the resistance to H_2_S poisoning.

## Conclusions

CO_2_ methanation was studied over catalysts composed of 13 wt.% of nickel and different supports based on graphene and alumina. Among graphene-based supports, the best option was the 13Ni/rGO catalyst because unlike 13Ni/AGO, 13Ni/BGO, 13Ni-Ol/GO, 13Ni/Ol-GO, or 13Ni/Ol-GO Met, it retains functional oxygen groups in an accessible way. These allow the anchoring of the active metal to its surface, reaching 78% methane yield values at 810 K and 10 bar.

The ability of graphene oxide-derived supports to conduct and dissipate the heat produced in the exothermic methanation reaction probably results in a slight decrease of the hot spot formation and a better uniformity in the temperature thorough the catalyst particle. However, in spite of avoiding hot spots in the catalyst, the progressive increase in temperature causes the rupture of oxygen bonds in the catalyst structure, causing the loss of active centers and loss of catalytic activity.

The use of graphene oxide-derived supports in methanation was not suitable, based on what has been observed in terms of activity or resistance to poisoning, since it did not improve the results obtained for the alumina-supported catalysts. A deep study of methods to stabilize these supports when temperature is increased should be performed.

Lanthanum interacts with nickel and alumina support, producing a reduction in nickel particle size (from 10 to 5 nm), which improves its dispersion. This, in turn, produces an increase in catalytic activity at lower temperatures (from 44 to 82% at 673 K) due to a synergetic effect on the catalytic activity of these metals. Finally, the incorporation of lanthanum results in the improvement of the resistance of the catalyst to H_2_S poisoning (from 70 to 110 min), making it difficult to block the active centers of the metal and by inhibiting the formation of the S^2−^ bond on the metal surface.

## Data Availability

Data and materials are provided in this manuscript. There is no supplementary information.
